# Supplementary material for: Single-cell landscape of nuclear configuration and gene expression during stem cell differentiation and X inactivation
Source: Genome Biol. 2021 Sep 27;22:279. doi: 10.1186/s13059-021-02432-w (PMC8474932; doi:10.1186/s13059-021-02432-w)
Supplement: Supplementary file 2 — Additional file 1: A file containing supplementary material including Supplementary Figures, Supplementary Tables, and a description of the Supplementary Video. [file 13059_2021_2432_MOESM1_ESM.pdf]

Supplementary material

Single-cell landscape of nuclear configuration and gene expression during stem cell differentiation and X inactivation

Giancarlo Bonora, Vijay Ramani, Ritambhara Singh, He Fang, Dana Jackson, Sanjay Srivatsan, Ruolan Qiu, Choli Lee, Cole Trapnell, Jay Shendure, Zhijun Duan, Xinxian Deng, William S. Noble, Christine M. Disteche

Table of contents

Supplementary Figures……………………………………………………………………………….………………...1

Supplementary Tables…………………………………………………………………………………………….……..16

Supplementary Video...……………………………………………………………………………………………….21

Supplementary Figures

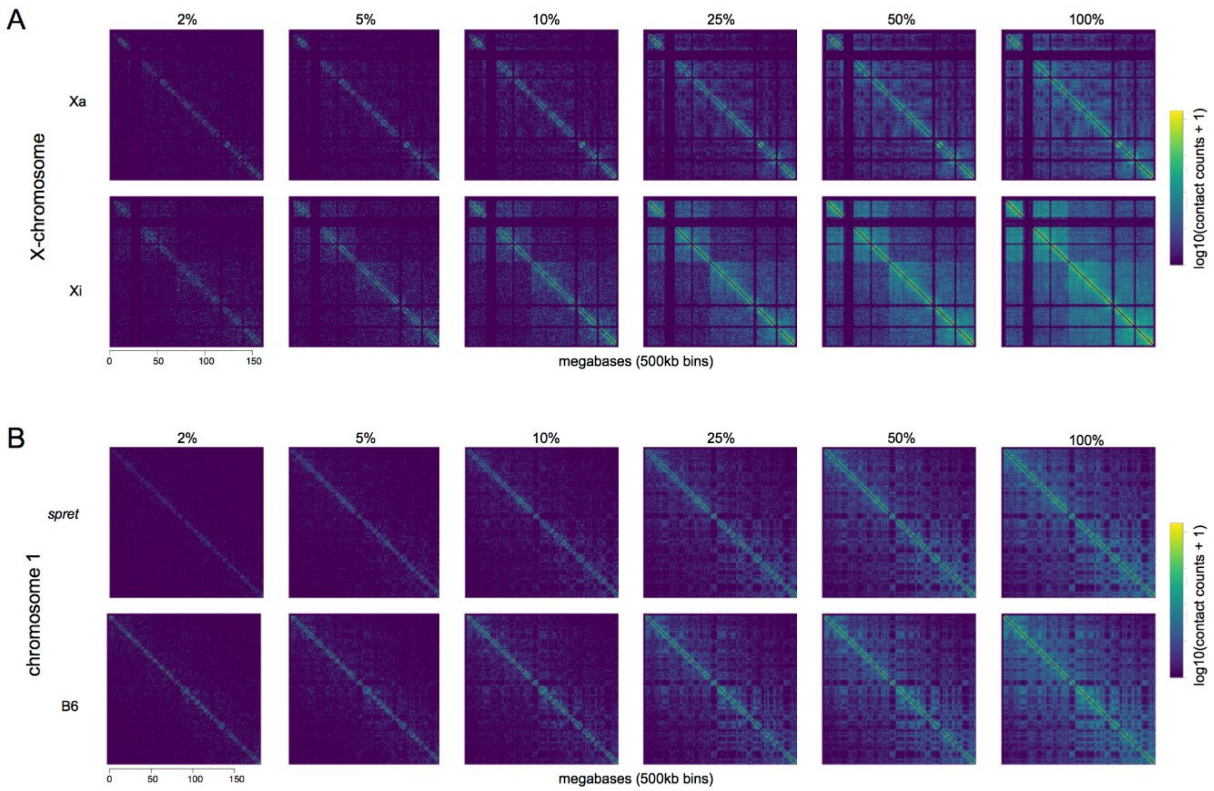

Fig. S1. Pseudobulk sci-Hi-C contact maps using different numbers of Patski cells.
A) Aggregate (pseudobulk) allelic contact maps of Xi and Xa using 2 – 100% (4,493 total cells) of randomly sampled available cells from the Patski sci-Hi-C experiment. Color scale reflects log contact counts.
B) As in A, but for maternal (B6) and paternal (*spret)* alleles of chromosome 1.

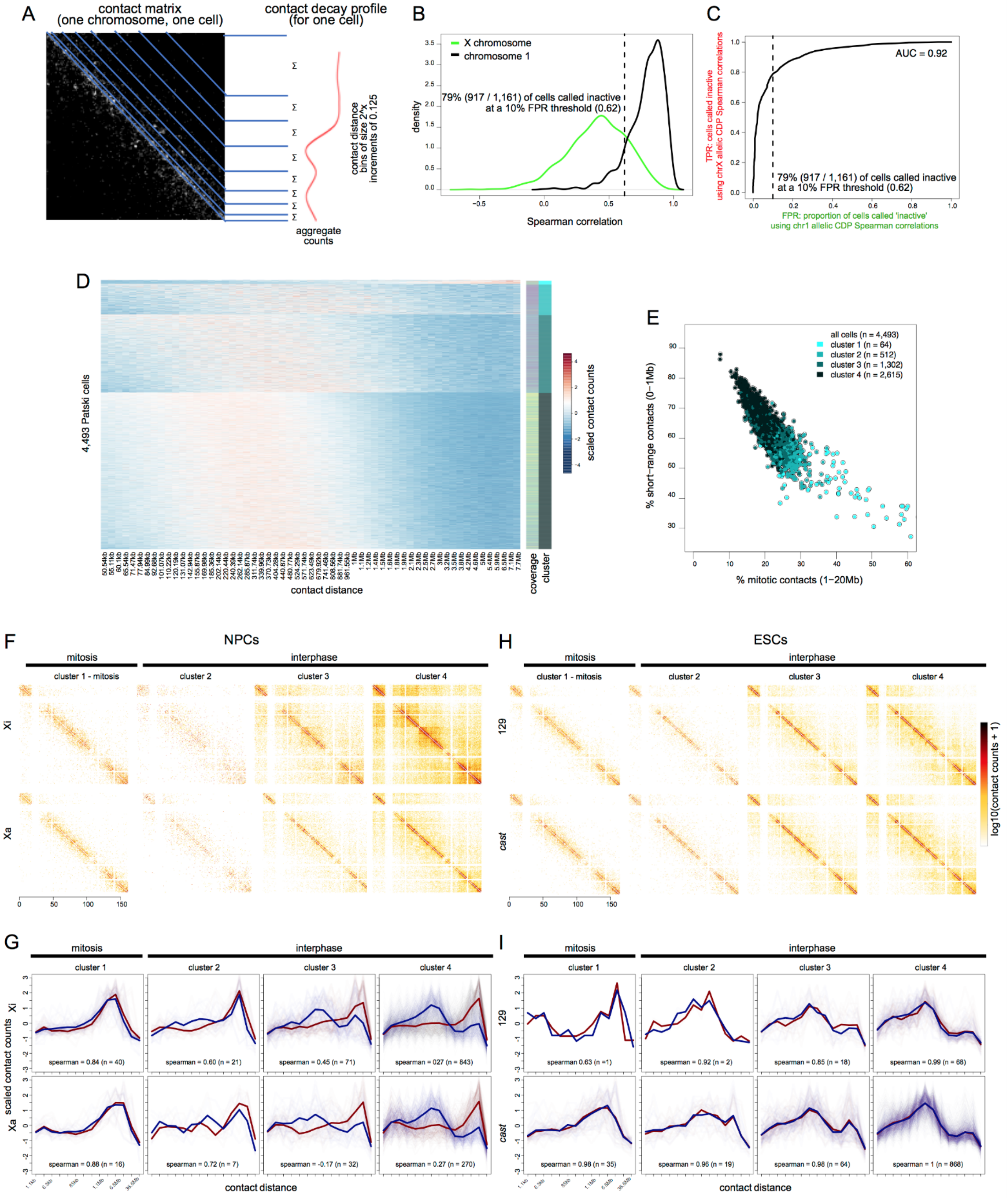
A
contact matrix
(one chromosome, one cell)
contact decay profile
(for one cell)
contact distance
bins of size 2^x
increments of 0.125
aggregate
counts
B
X chromosome
chromosome 1
79% (917 / 1,161) of cells called inactive
at a 10% FPR threshold (0.62)
density
Spearman correlation
C
AUC = 0.92
79% (917 / 1,161) of cells called inactive
at a 10% FPR threshold (0.62)
TPR: cells called inactive
using chrX allelic CDP Spearman correlations
FPR: proportion of cells called 'inactive'
using chr1 allelic CDP Spearman correlations
D
4,493 Patski cells
scaled contact counts
contact distance
coverage
cluster
E
all cells (n = 4,493)
cluster 1 (n = 64)
cluster 2 (n = 512)
cluster 3 (n = 1,302)
cluster 4 (n = 2,615)
% short−range contacts (0−1Mb)
% mitotic contacts (1−20Mb)
F
NPCs
mitosis
interphase
cluster 1 - mitosis
cluster 2
cluster 3
cluster 4
Xi
Xa
H
ESCs
mitosis
interphase
cluster 1 - mitosis
cluster 2
cluster 3
cluster 4
129
cast
log10(contact counts + 1)
G
mitosis
interphase
cluster 1
cluster 2
cluster 3
cluster 4
Xi
Xa
scaled contact counts
spearman = 0.84 (n = 40)
spearman = 0.60 (n = 21)
spearman = 0.45 (n = 71)
spearman = 027 (n = 843)
spearman = 0.88 (n = 16)
spearman = 0.72 (n = 7)
spearman = -0.17 (n = 32)
spearman = 0.27 (n = 270)
contact distance
I
mitosis
interphase
cluster 1
cluster 2
cluster 3
cluster 4
129
cast
spearman = 0.63 (n =1)
spearman = 0.92 (n = 2)
spearman = 0.85 (n = 18)
spearman = 0.99 (n = 68)
spearman = 0.98 (n = 35)
spearman = 0.96 (n = 19)
spearman = 0.98 (n = 64)
spearman = 1 (n = 868)
contact distance

Fig. S2. Strategy to determine CDPs, and cell cycle analyses in Patski cells, NPCs, and ESCs.

A) Example of a contact map for a single chromosome of a single cell based on sci-Hi-C data overlaid with a schematic of exponentially-increasing contact distances ranges used to summate contact counts with respect to distance in order to produce contact decay profiles (CDPs), shown to the right.

B) Distributions of Spearman correlations between the CDPs of each allele for each of 1,161 cells for chrX (green; as described in Fig. 2B) and chr1 (black; as described in Fig. 2F). The dashed line represents a threshold chosen below which cells were called as having a bipartite Xi. This threshold represents a 10% false positive rate (FPR) based on the distribution allelic CDP Spearman correlations for chr1 (see D).

C) Plot of true positive rate (TPR; proportion of Patski cells called as having a bipartite Xi based on the Spearman correlations between allelic CDPs for chrX as described in Fig. 2B, C) versus false positive rate (FPR; proportion of Patski cells called 'inactive' based on the Spearman correlations between allelic CDPs for chr1 as described in Fig. 2F, G) at all thresholds of Spearman correlation value (0 to 1). The area under the curve (AUC) is 0.92. The dashed line represents the 10% FPR threshold chosen below which cells were called as having a bipartite Xi based on chrX allelic CDP Spearman correlation.

D) Heatmaps of biallelic CDPs for the autosomes of 4,493 Patski cells clustered into four groups using *k*-means clustering and the Spearman correlation distance between the cells' CDPs. Heatmap colors reflect z-scaled counts within logarithmic bins with the left-hand boundaries specified along the bottom from 50 kb to 8 Mb.

E) Scatter plot of the proportion of short-range contact pairs (within 1 Mb) versus mitotic contact pairs (between 1 and 20 Mb) for 4,493 Patski cells. Cells colored by the *k*-means clusters described in C**.**

F) Aggregate allelic contact maps for the Xi and Xa using contact pairs from cells grouped using the k-means clustering of the autosomal CDPs of F121 and Tsix-stop NPCs as described in Fig. S2D.

G) Plots of allelic CDPs for the Xi (red) and Xa (blue) for cells in each of the four clusters described in F. The average CDP is plotted over plots of the CDPs for each individual cell. The Spearman correlations between the average allelic CDPs is given at the bottom of the plot, along with cell counts used in the analysis.

H, I) As in F, G, respectively, but for the 129 and *cast* alleles of F121 and ES_Tsix-stop d0 cells (ESCs).

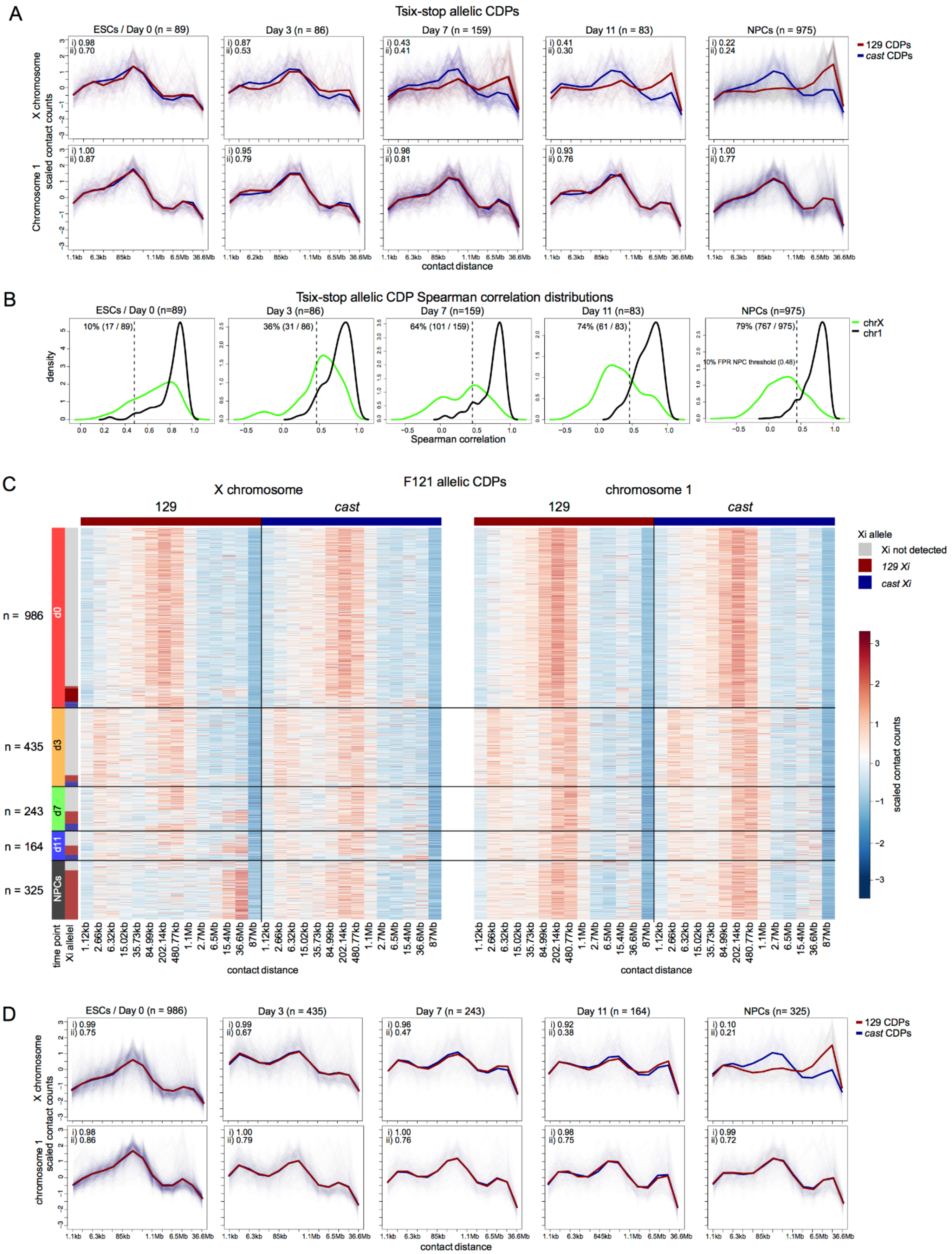
A
Tsix-stop allelic CDPs
ESCs / Day 0 (n = 89)
Day 3 (n = 86)
Day 7 (n = 159)
Day 11 (n = 83)
NPCs (n = 975)
129 CDPs
cast CDPs
X chromosome
Chromosome 1
scaled contact counts
contact distance
B
Tsix-stop allelic CDP Spearman correlation distributions
ESCs / Day 0 (n=89)
Day 3 (n=86)
Day 7 (n=159)
Day 11 (n=83)
NPCs (n=975)
10% (17 / 89)
36% (31 / 86)
64% (101 / 159)
74% (61 / 83)
79% (767 / 975)
10% FPR NPC threshold (0.48)
chrX
chr1
density
Spearman correlation
C
F121 allelic CDPs
X chromosome
chromosome 1
129
cast
Xi allele
Xi not detected
129 Xi
cast Xi
scaled contact counts
n = 986
n = 435
n = 243
n = 164
n = 325
d0
d3
d7
d11
NPCs
time point
Xi allele
contact distance
D
ESCs / Day 0 (n = 986)
Day 3 (n = 435)
Day 7 (n = 243)
Day 11 (n = 164)
NPCs (n= 325)
129 CDPs
cast CDPs
X chromosome
chromosome 1
scaled contact counts
contact distance

Fig. S3. Tsix-stop CDPs and F121 heatmaps and CDPs for chr X and chr1 during differentiation.

A) Plots of allelic contact decay profiles (CDPs) for Tsix-stop cells with at least 50 contacts per allele within chrX (Xi, red; Xa, blue;) and chr1 (129, red; *cast*, blue;) for the indicated time points during differentiation from ESCs (d0) to EBs (day 11), as well as differentiated NPCs. The plots show z-scaled counts within contact distance ranges along the x-axis. Contact counts were binned within exponentially-increasing contact distances ranges (2^x where x was incremented by 0.125). These bins were then aggregated further to reduce noise by combining the counts within 10 non-overlapping bins at a time. The number of cells at each time point is given by the n number. The average allelic CDP is plotted over the plots of CDPs for each individual cell. Spearman correlation values between the two mean allelic CDPs (i) as well as the median of the Spearman correlation values between the allelic CDPs for each individual cell (ii) are given in the top left corner of each plot.

B) Distributions of Spearman correlations between CDPs of each allele for each of the cells as in A for chrX (green) and chr1 (black). The dashed line represents a threshold chosen below which cells were called having a bipartite Xi based on the chrX allelic CDP Spearman correlation. This threshold represents a 10% false positive rate (FPR) based on the distribution for chr1 allelic CDP Spearman correlations.

C) As in Fig. 3A, but for 2,153 F121 cells. (Note that XCI is skewed towards the 129 allele in the NPCs.)

D) As in A, but for F121 cells.

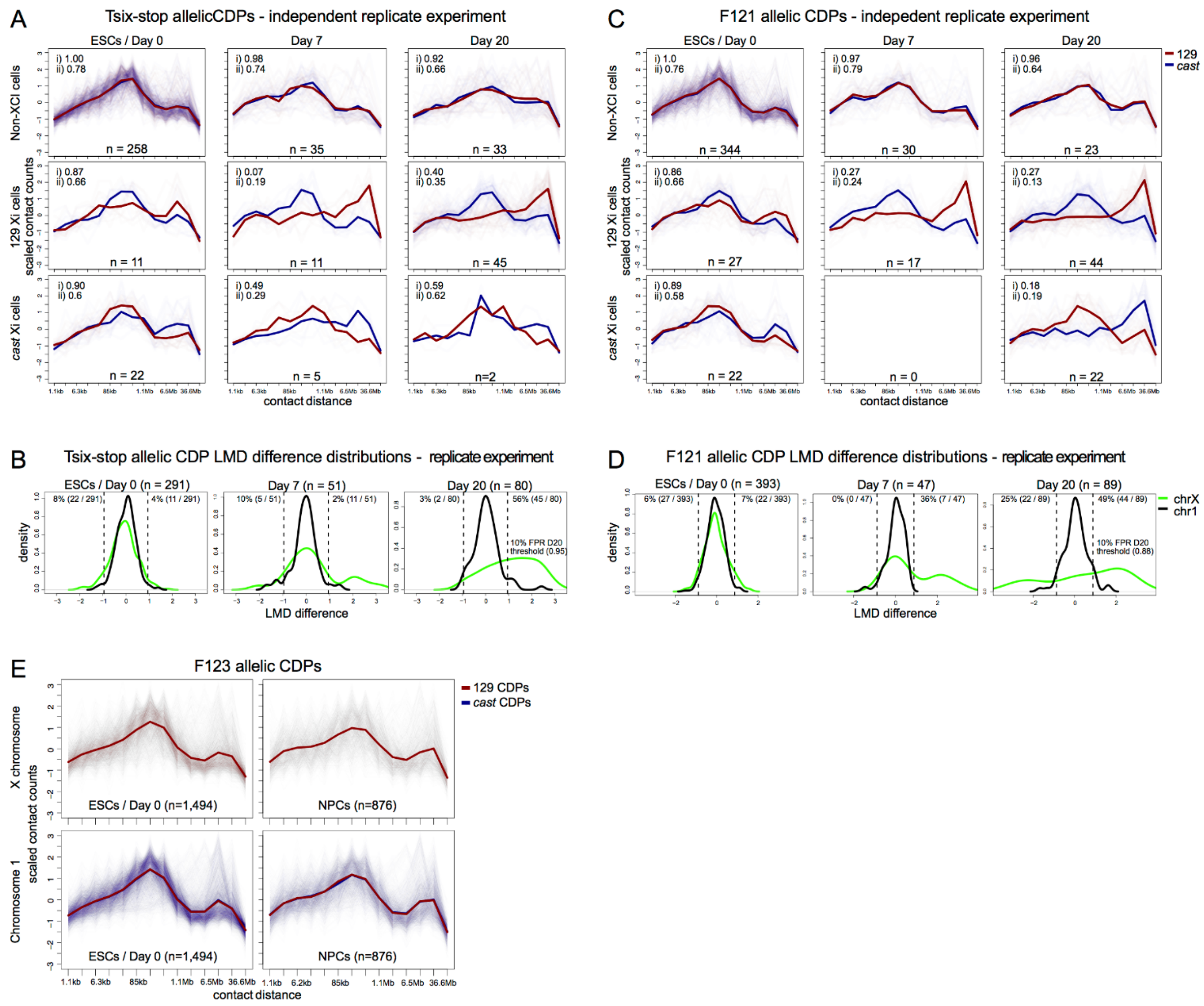

Fig. S4. CDPs for replicate Tsix-stop and F121 sci-Hi-C experiments and for F123 cells.

A) Plots of allelic CDPs for Tsix-stop for the indicated time points during differentiation from d0 to d7 and d20 (beyond embryoid bodies) for cells classified as not having a bipartite Xi (top row), and cells that do exhibit CDPs indicative of an bipartite Xi on their 129 allele (middle row; XCI is skewed towards the 129 allele) or their *cast* allele (bottom row; XCI is skewed towards the *cast* allele). Only cells with at least 50 contacts per allele within both chrX and chr1. The plots show z-scaled counts within contact distance ranges shown along the x-axis. Contact counts were binned within exponentially-increasing contact distances ranges (2^x where x was incremented by 0.125). These bins were then aggregated further to reduce noise by combining the counts within 10 non-overlapping bins at a time. The number of cells at each time point is given by the n number. The average allelic CDP is plotted over the plots of CDPs for each individual cell. Spearman correlation values between the two mean allelic CDPs (i) as well as the median of the Spearman correlation values between the allelic CDPs for each individual cell (ii) are given in the top left corner of each plot.

B) Distributions of the difference between the long-range to mid-range differences (LMDs) of each allele (Fig. 2I and Methods) for each of the cells as described in A for chrX (green) and chr1 (black). The dashed lines represent a threshold chosen below which cells were called as having a bipartite Xi based on chrX allelic CDP LMD differences. This threshold represents a 10% false positive rate (FPR) based on the distribution for chr1 allelic CDP LMD differences.

C) As in A, but for F121 cells.

D) As in B, but for F121 cells.
E) As in Fig. S3A, but for F123 ESCs and NPCs. Note that these male cells only possess a 129 chrX.

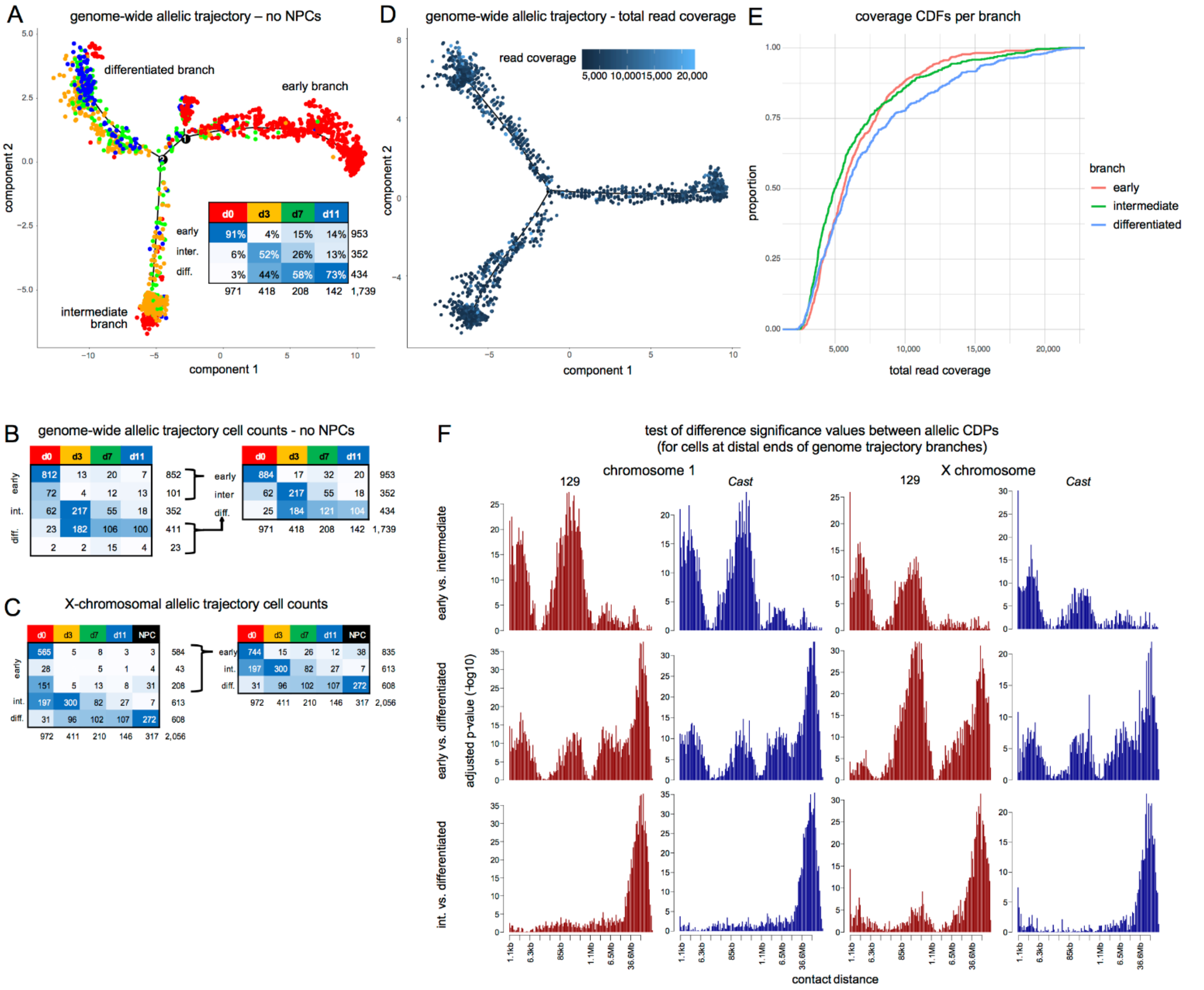

Fig. S5. Trajectory analysis of F121 cells based on allelic CDPs.
A) As in Fig. 5A, but excluding NPCs and based on 2,199 significantly differential bins (q-value <= 10^{-9}).
B) Counts per time point per trajectory branch shown in A before and after collapsing two early and two differentiated sub-branches into a single early branch and a single differentiated branche, respectively.
C) As in B but for the X-chromosome allelic CDP trajectory in Fig. 5C before and after collapsing three early sub-branches into a single branch.
D) As in Fig. 5A, but with cells colored by total contact counts.
E) Cumulative distribution functions (CDFs) of the total contact counts per cells along each branch of the trajectory shown in Fig. 5A.
F) Plots of the -log10 adjusted p-values from t-tests between the values within every logarithmic bin (2^x where x was incremented by 0.125) of the allelic chr1 and chrX CDPs (129, red; *cast,* blue) for cells at the distal ends of each pair of branches (rows) in the trajectory described in Fig. 5E, F.

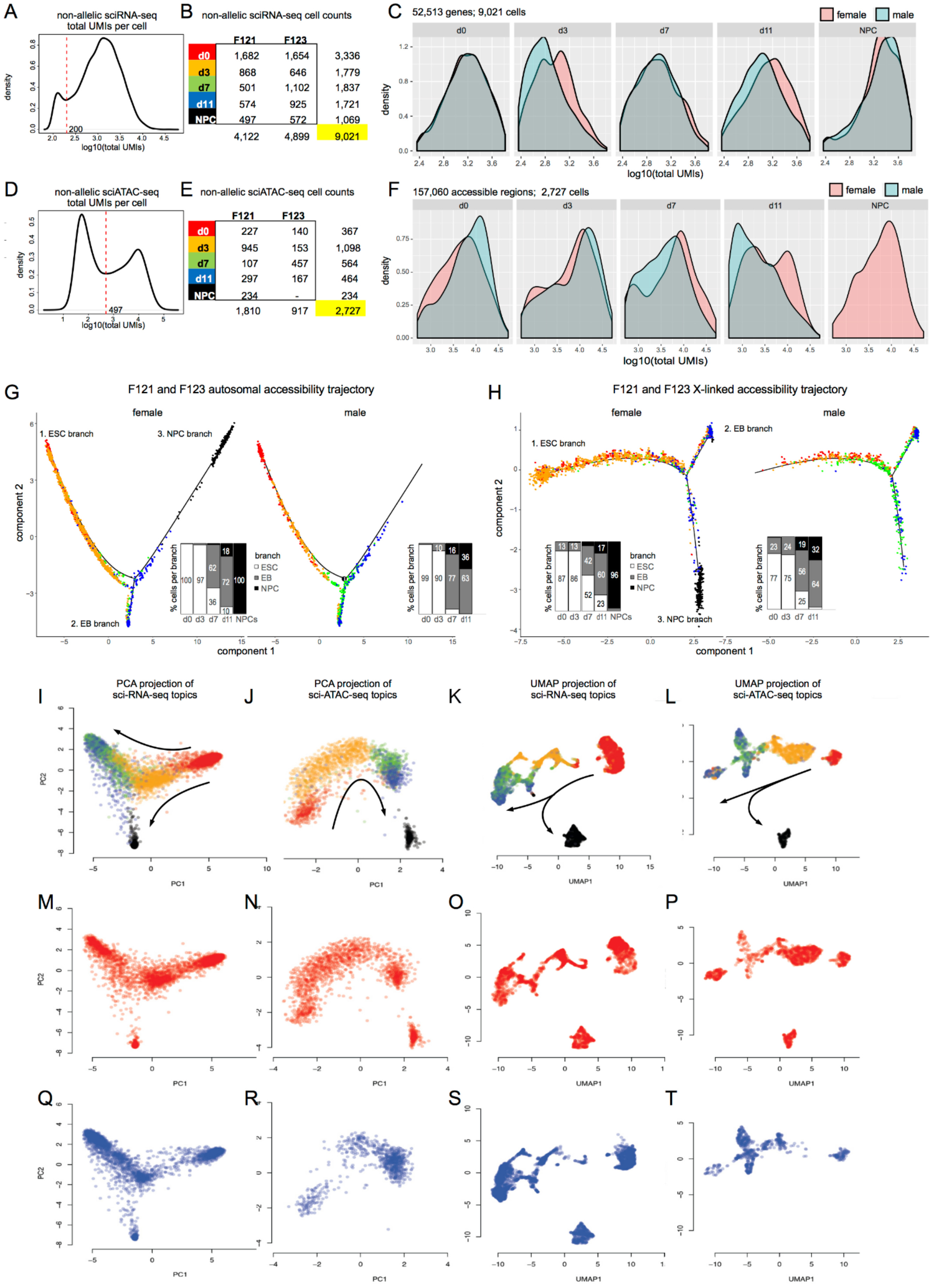
A
non-allelic sciRNA-seq
total UMIs per cell
density
200
log10(total UMIs)
B
non-allelic sciRNA-seq cell counts
F121 F123
d0 1,682 1,654 3,336
d3 868 646 1,779
d7 501 1,102 1,837
d11 574 925 1,721
NPC 497 572 1,069
4,122 4,899 9,021
C
52,513 genes; 9,021 cells
female male
d0 d3 d7 d11 NPC
density
log10(total UMIs)
D
non-allelic sciATAC-seq
total UMIs per cell
density
497
log10(total UMIs)
E
non-allelic sciATAC-seq cell counts
F121 F123
d0 227 140 367
d3 945 153 1,098
d7 107 457 564
d11 297 167 464
NPC 234 - 234
1,810 917 2,727
F
157,060 accessible regions; 2,727 cells
female male
d0 d3 d7 d11 NPC
density
log10(total UMIs)
G
F121 and F123 autosomal accessibility trajectory
female
male
1. ESC branch
3. NPC branch
2. EB branch
% cells per branch
branch
ESC
EB
NPC
d0 d3 d7 d11 NPCs
component 2
component 1
H
F121 and F123 X-linked accessibility trajectory
female
male
1. ESC branch
2. EB branch
3. NPC branch
% cells per branch
branch
ESC
EB
NPC
d0 d3 d7 d11 NPCs
component 2
component 1
I
PCA projection of
sci-RNA-seq topics
J
PCA projection of
sci-ATAC-seq topics
K
UMAP projection of
sci-RNA-seq topics
L
UMAP projection of
sci-ATAC-seq topics
PC1
PC2
UMAP1
M
N
O
P
Q
R
S
T

Fig. S6. Sex differences in non-allelic expression and accessibility data distribution, and topic modeling projections.

A) Distributions of total genome-wide expression (log10[UMIs]) from sci-RNA-seq in F121 and F123 cells. The vertical line shows the ~200 UMI minima used as a threshold to filter out cells with low expression.
B) Table of F121 and F123 cell counts per time point after applying the filter described in A.
C) Distributions of total genome-wide expression (log10[UMIs]) from sci-RNA-seq in F121 and F123 cells faceted by time point in cells showing a minimum total expression of 200 UMI.
D) As in A, but based on total genome-wide chromatin accessibility (log10[UMIs]) from sci-ATAC-seq in F121 and F123 cells. The vertical line shows the ~500 UMI minima used as a threshold to filter out cells with low values.
E) As in B, but based on total genome-wide accessibility (log10[UMIs]) from sci-ATAC-seq in F121 and F123 cells.
F) Distributions of total genome-wide accessibility (log10[UMIs]) from sci-ATAC-seq in F121 and F123 cells faceted by time point in cells showing a minimum total accessibility counts of 500 UMI.
G) As in Fig. 6C, but based on chromatin accessibility data from sci-ATAC-seq for 2,919 of the top 25% most variable autosomal regions showing coverage in at least 10 cells and significantly differential across time points (q-value $<= 10^{-21}$).
H) As in G, but for 4,359 regions along chrX accessible in at least 10 cells and significantly differential across time points (q-value <= 0.01).
I) Projection using the first two components from PCA of a topic matrix generated from non-allelic F121 and F123 sci-RNA-seq data. The matrix of topics per cell was decomposed from a matrix of UMI counts within genes for each cell. Each data point represents a cell colored by time point. Arrows indicate the direction of development.
J) As in I, but for non-allelic sci-ATAC-seq data. The matrix of topics per cell was decomposed from a matrix of UMI counts within accessible regions for each cell.
K) As in I, but using UMAP.
L) As in J, but using UMAP.
M–P) As in I–L, but showing F121 cells only.
Q–T) As in I–L, but showing F123 cells only.

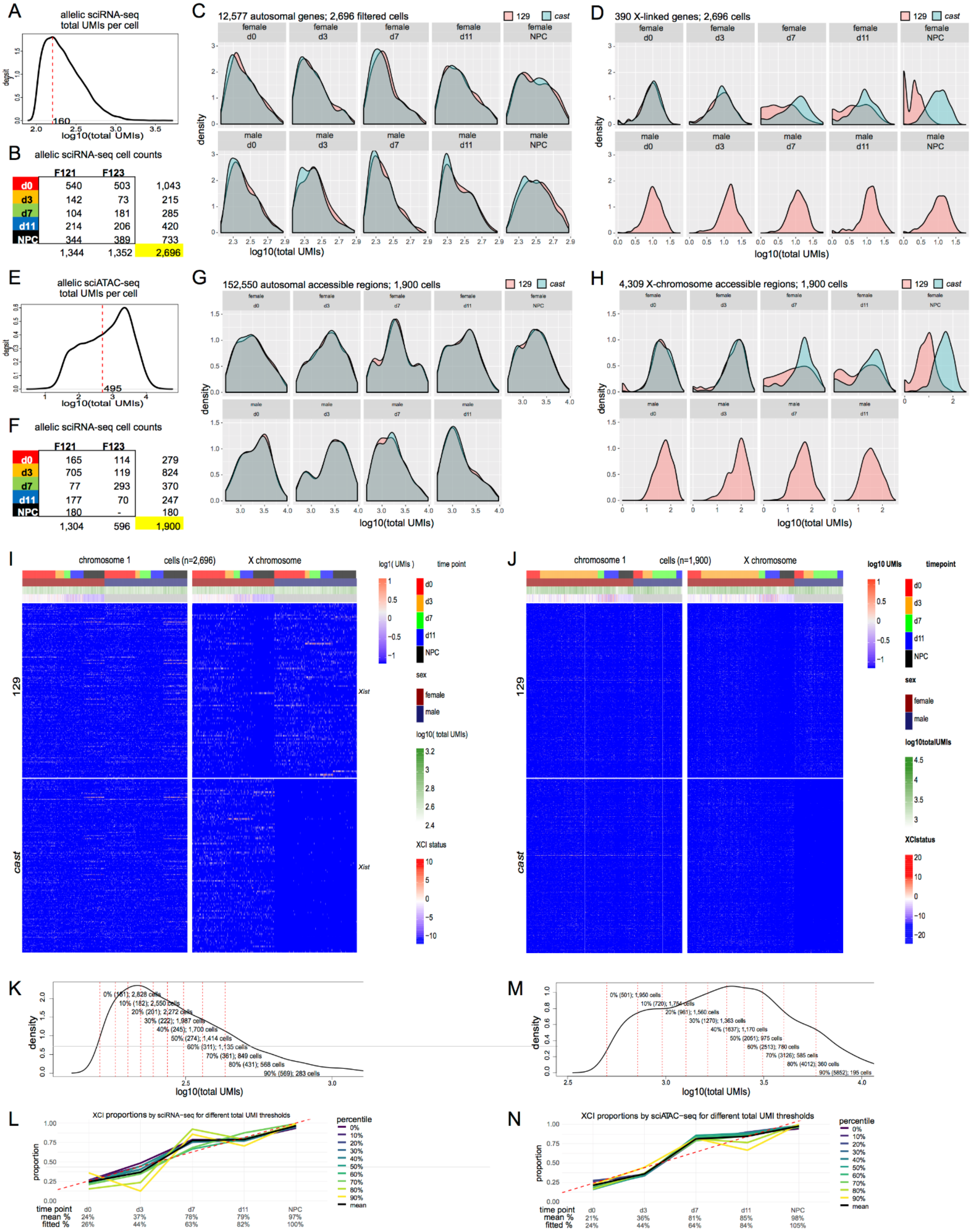

A
allelic sciRNA-seq total UMIs per cell
160
log10(total UMIs)
B
allelic sciRNA-seq cell counts
F121 F123
d0 540 503 1,043
d3 142 73 215
d7 104 181 285
d11 214 206 420
NPC 344 389 733
1,344 1,352 2,696
C
12,577 autosomal genes; 2,696 filtered cells
129 cast
female d0 female d3 female d7 female d11 female NPC
male d0 male d3 male d7 male d11 male NPC
density
log10(total UMIs)
D
390 X-linked genes; 2,696 cells
E
allelic sciATAC-seq total UMIs per cell
495
log10(total UMIs)
F
allelic sciRNA-seq cell counts
F121 F123
d0 165 114 279
d3 705 119 824
d7 77 293 370
d11 177 70 247
NPC 180 - 180
1,304 596 1,900
G
152,550 autosomal accessible regions; 1,900 cells
H
4,309 X-chromosome accessible regions; 1,900 cells
I
chromosome 1
cells (n=2,696)
X chromosome
129
cast
Xist
log1(UMIs)
time point
sex
female
male
log10(total UMIs)
XCI status
J
chromosome 1
cells (n=1,900)
X chromosome
log10 UMIs
timepoint
log10totalUMIs
XCIstatus
K
0% (161); 2,828 cells
10% (182); 2,550 cells
20% (201); 2,272 cells
30% (222); 1,987 cells
40% (245); 1,700 cells
50% (274); 1,414 cells
60% (311); 1,135 cells
70% (361); 849 cells
80% (431); 568 cells
90% (569); 283 cells
density
log10(total UMIs)
M
0% (501); 1,950 cells
10% (720); 1,754 cells
20% (961); 1,560 cells
30% (1270); 1,363 cells
40% (1637); 1,170 cells
50% (2051); 975 cells
60% (2513); 780 cells
70% (3126); 585 cells
80% (4012); 360 cells
90% (5852); 195 cells
L
XCI proportions by sciRNA-seq for different total UMI thresholds
proportion
percentile
0% 10% 20% 30% 40% 50% 60% 70% 80% 90% mean
time point d0 d3 d7 d11 NPC
mean % 24% 37% 78% 79% 97%
fitted % 26% 44% 63% 82% 100%
N
XCI proportions by sciATAC-seq for different total UMI thresholds
time point d0 d3 d7 d11 NPC
mean % 21% 36% 81% 85% 98%
fitted % 24% 44% 64% 84% 105%

Fig. S7. Allelic expression and accessibility distributions, filtering, and visualization.
A) Distributions of total genome-wide allelic expression (log10[UMIs]) from sci-RNA-seq in F121 and F123 cells. The vertical line shows the ~160 UMI maxima used as a threshold to filter out cells with low expression. This value was used since no minima was available in the distribution to use as a threshold.
B) Table of F121 and F123 cell counts per time point after applying the filter described in A.
C) Allelic expression (log10[UMIs]) distributions based on sci-RNA-seq data for autosomal genes faceted by time point (columns) and sex (rows).
D) As in C, but for expression distributions based on X-linked genes.
E) As in A, but based on total genome-wide accessibility (log10[UMIs]) from sci-ATAC-seq in F121 and F123 cells. The vertical line shows the ~500 UMI value at an inflection point in the distribution used as a threshold to filter out cells with low expression. This value was used since no minima was available in the distribution to use as a threshold and it closely matched the value used for non-allelic data.
F) As in B, but based on total genome-wide allelic accessibility (log10[UMIs]) from sci-ATAC-seq on F121 and F123 cells.
G) As in C, but for accessibility based on allelic sci-ATAC-seq data.
H) As in G, but for accessibility distributions based on accessible regions along chrX.
I) Heatmap of allelic expression (log UMIs) as measured by sci-RNA-seq for chr1 (left) and chrX (right) for the 25% most variability expressed genes per chromosome (rows) for 2,696 F121 and F123 cells that passed filter. Cells sorted by annotations described in legend on the right of the heatmaps.
J) As in I, but for allelic accessibility as measured by sci-ATAC-seq 25% most variability accessible regions per chromosome (rows) for 1,900 F121 and F123 cells that passed filter.
K) Distributions of total allelic UMIs per cell for sci-RNA-seq. Vertical lines represent quantile thresholds used in robustness testing results plotted in L.
L) Plots of the proportion (%) of F121 cells with a silenced chrX using sci-RNA-seq expression and the method described in Fig. 8C for different proportions of cells based on total expression quantiles. This shows that the proportions of cells showing XCI silencing based on allelic sci-RNA-seq is robust to coverage level and cell numbers.
M) As in K, but based on sci-ATAC-seq accessibility data for robustness testing results plotted in N.
N) Plots of the proportion (%) of F121 cells with a low accessibility X chromosome data using sci-ATAC-seq accessibility data and the method described in Fig. 8D for different proportions of cells based on total accessibility quantiles. This shows that the proportions of cells that have undergone XCI based on allelic sci-ATAC-seq is robust to coverage level and cell numbers.

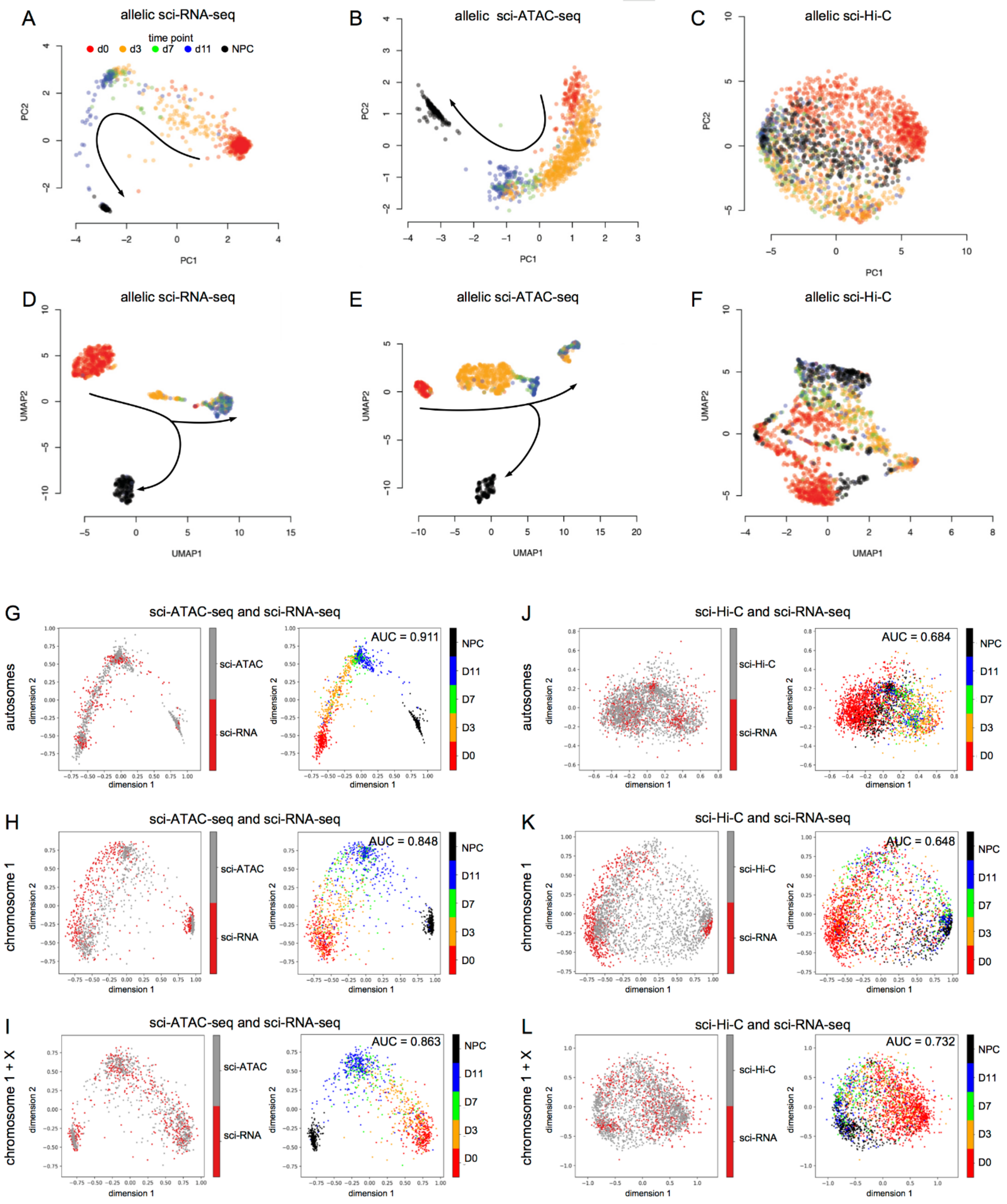
A
allelic sci-RNA-seq
time point
d0
d3
d7
d11
NPC
PC1
PC2
B
allelic sci-ATAC-seq
C
allelic sci-Hi-C
D
allelic sci-RNA-seq
UMAP1
UMAP2
E
allelic sci-ATAC-seq
F
allelic sci-Hi-C
G
sci-ATAC-seq and sci-RNA-seq
autosomes
sci-ATAC
sci-RNA
AUC = 0.911
NPC
D11
D7
D3
D0
dimension 1
dimension 2
H
sci-ATAC-seq and sci-RNA-seq
chromosome 1
AUC = 0.848
I
sci-ATAC-seq and sci-RNA-seq
chromosome 1 + X
AUC = 0.863
J
sci-Hi-C and sci-RNA-seq
autosomes
sci-Hi-C
sci-RNA
AUC = 0.684
K
sci-Hi-C and sci-RNA-seq
chromosome 1
AUC = 0.648
L
sci-Hi-C and sci-RNA-seq
chromosome 1 + X
AUC = 0.732

Fig. S8. Projections of allelic topic matrices used for multimodal alignment.
A) Projection using the first two components from PCA of a topic matrix generated from allelic sci-RNA-seq data that served as input to the MMD-MA algorithm. The matrix of topics per cell was decomposed from a matrix of UMI counts within genes from each allele concatenated together (rows) for each cell (columns). Each data point represents a cell colored by time point. Arrows indicate the direction of development.
B) As in A, but for allelic sci-ATAC-seq data. The matrix of topics per cell was decomposed from a matrix of UMI counts within accessible regions from each allele concatenated together (rows) for each cell (columns).
C) As in A, but for allelic sci-Hi-C data. The matrix of topics per cell was decomposed from a matrix of concatenated contact decay counts within logarithmically-increasing sized bins for both alleles of each chromosome (rows) for each cell (columns).
D) As in A, but using UMAP.
E) As in B, but using UMAP.
F) As in C, but using UMAP.
G) As in Fig. 9A, but only using autosomal data.
H) As in Fig. 9A, but only using chr1 data.
I) As in Fig. 9A, but only using data from chr1 and chrX.
J) As in Fig. 9B, but only using autosomal data.
K) As in Fig. 9B, but only using chr1 data.
L) As in Fig. 9B, but only using data from chr1 and chrX.

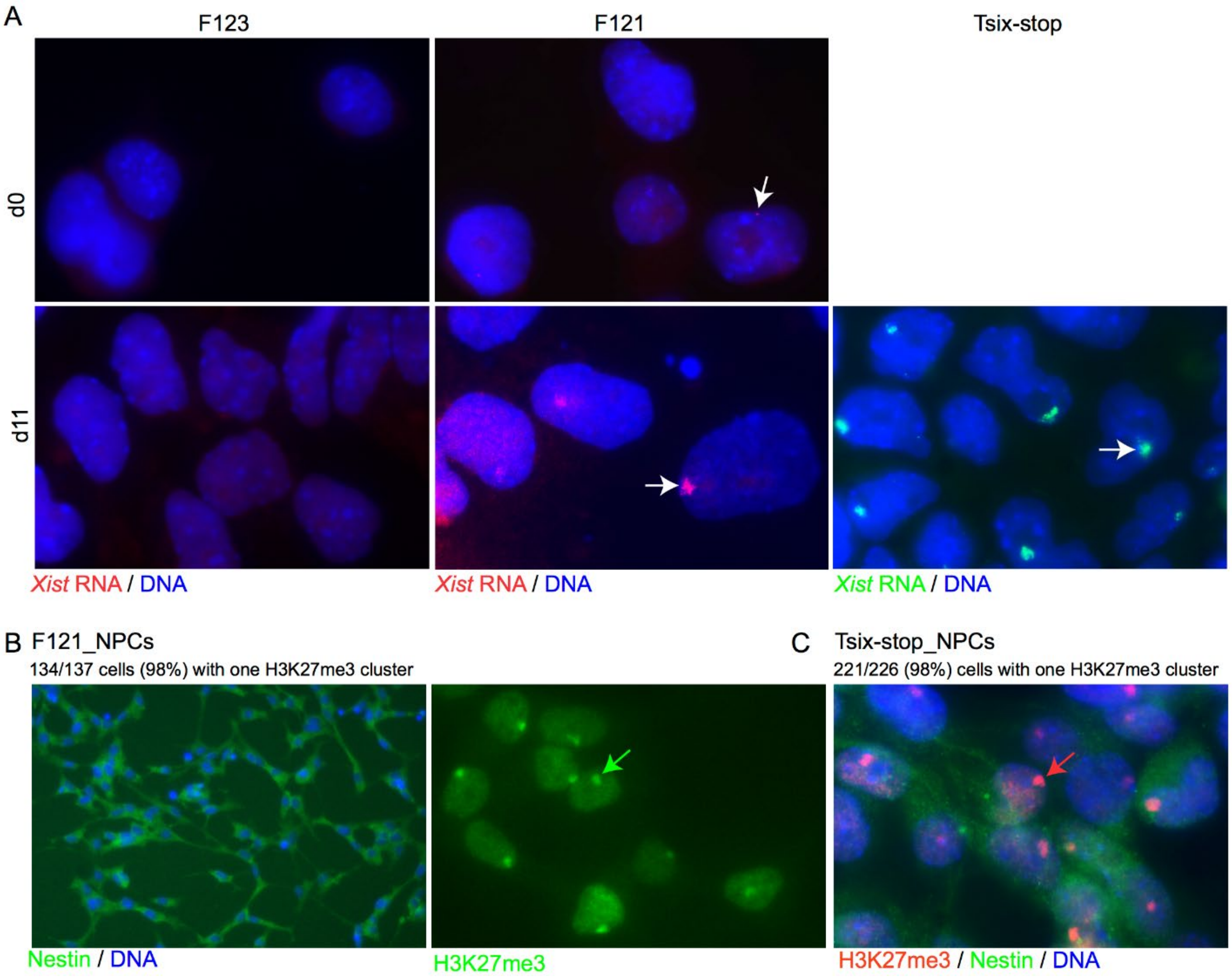

Fig. S9. Verification of establishment of XCI during in vitro differentiation of mESCs.

A) RNA-FISH using an *Xist* probe in male and female d0 and d11 cells show the presence of an *Xist* cloud (arrows) in d11 differentiated cells from the female lines F121 and ES_Tsix-stop, but not the male F123 cells or d0 F121 female ESCs. 91/96 (95%) of Tsix-stop d11 cells show one *Xist* cloud, indicating the establishment of XCI. Note that some d0 cells show pinpoints of signals (arrowhead), indicating low levels of *Xist* expression in undifferentiated mESCs. *Xist* RNA-FISH was done using green or red fluorescence-labeled probes using a 10 kb *Xist* cDNA plasmid (pXho, which contains most of *Xist* exon 1). DNA is stained by Hoechst 33258 (blue).

B) Immunostaining using a H3K27me3 antibody detected by a FITC-conjugated secondary antibody (green) was done to mark the Xi in NPCs differentiated from F121. DNA is stained by Hoechst 33258 (blue). Immunostaining for nestin with an Alexa Fluor 488-conjugated antibody (green) confirmed differentiation into NPCs. 134/137 (98%) NPCs were scored as having one H3K27me3 cluster, indicating the establishment of XCI.

C) Co-immunostaining with a H3K27me3 antibody detected by a Texas red-conjugated secondary antibody (red) to mark the Xi and an Alexa Fluor 488-conjugated antibody for nestin (green) was done in Tsix-stop NPCs. DNA is stained by Hoechst 33258 (blue). 221/226 (98%) NPCs were scored as having one H3K27me3 cluster, indicating the establishment of XCI.

Supplementary Tables

Table S1. sci-Hi-C cell counts and non-allelic and allelic contact count summary statistics.

		Non-allelic interaction summary statistics					
		Genome-wide			X-chromosome		
sample	cell counts	total contacts	mean contacts	median contacts	total contacts	mean contacts	median contacts
Patski	4,493	39,289,629	8,745	5,167	2,153,390	479	280
TsixStop_d0	342	1,770,062	5,176	4,491	85,204	249	223
TsixStop_d3	839	2,878,080	3,430	2,785	134,247	160	128
TsixStop_d7	822	4,748,683	5,777	4,218	232,140	283	199
TsixStop_d11	471	3,517,515	7,468	3,392	176,193	374	153
TsixStop_NPC	2,535	16,481,251	6,501	4,903	834,206	329	246
F121_d0	2,203	17,874,237	8,114	6,245	890,230	404	305
F121_d3	894	9,688,584	10,837	6,297	578,606	647	367
F121_d7	732	10,684,487	14,596	6,866	494,258	675	302
F121_d11	608	7,266,390	11,951	6,064	314,722	518	260
F121_NPC	1,054	6,369,538	6,043	4,063	338,132	321	214
F123_d0	1,670	14,520,800	8,695	6,668	421,215	252	190
F123_NPC	1,030	6,662,438	6,468	5,167	192,234	187	152
replicate TsixStop_d0	795	8,777,503	11,041	6,339	388,210	488	262
replicate TsixStop_d7	152	1,751,101	11,520	7,638	74,368	493	322
replicate TsixStop_d20	404	5,348,989	13,240	3,951	257,677	638	189
replicate F121_d0	1,479	8,469,522	5,727	3,707	455,156	308	200
replicate F121_d7	89	2,279,421	25,611	7,739	111,292	1,250	382
replicate F121_d20	301	3,144,889	10,448	4,643	158,900	530	227

		Allelic interaction summary statistics						
		Genome-wide				X-chromosome		
sample	cell counts	B6/129 contacts	spretus/Cast contacts	total allelic contacts	% allelic contacts	B6/129 contacts	spretus/Cast contacts	total allelic contacts
Patski	4,493	15,141,925	11,144,006	26,285,931	67%	714,755	575,179	1,289,934
TsixStop_d0	342	560,583	528,075	1,088,658	62%	17,984	18,977	36,961
TsixStop_d3	839	907,602	855,558	1,763,160	61%	27,939	30,936	58,875
TsixStop_d7	822	1,478,343	1,397,457	2,875,800	61%	48,383	56,053	104,436
TsixStop_d11	471	1,096,305	1,028,002	2,124,307	60%	36,138	42,144	78,282
TsixStop_NPC	2,535	5,192,202	4,853,045	10,045,247	61%	185,676	182,927	368,603
F121_d0	2,203	5,732,612	5,069,354	10,801,966	60%	200,624	186,690	387,314
F121_d3	894	2,875,116	2,576,480	5,451,596	56%	121,324	114,033	235,357
F121_d7	732	3,161,922	2,869,610	6,031,532	56%	89,467	113,557	203,024
F121_d11	608	2,148,784	1,957,360	4,106,144	57%	55,715	72,183	127,898
F121_NPC	1,054	2,051,798	1,818,259	3,870,057	61%	74,618	75,030	149,648
F123_d0	1,670	4,757,858	4,115,468	8,873,326	61%	175,901	12,429	188,330
F123_NPC	1,030	2,214,102	1,874,402	4,088,504	61%	80,332	5,816	86,148
replicate TsixStop_d0	795	2,629,500	2,428,991	5,058,491	58%	76,029	85,089	161,118
replicate TsixStop_d7	152	519,105	485,130	1,004,235	57%	12,194	18,585	30,779
replicate TsixStop_d20	404	1,572,477	1,506,863	3,079,340	58%	48,830	60,170	109,000
replicate F121_d0	1,479	2,597,383	2,309,070	4,906,453	58%	98,774	89,158	187,932
replicate F121_d7	89	688,096	621,063	1,309,159	57%	21,981	24,050	46,031
replicate F121_d20	301	943,181	849,736	1,792,917	57%	34,696	32,453	67,149

Counts of cells with at least 1,000 uniquely mapped contact pairs, a *cis:trans* ratio ≥1 and ≥95% of reads mapping to either the mouse or human genome (see Methods) for each sci-Hi-C library, along with the total, mean, and median number of contact pairs per cell genome-wide and along chrX. The number of allelic contacts for each allele and in total is also provided for the genome (along the percentage of non-allelic contacts) and chrX.

Table S2. Counts and percentages of cells showing XCI by allelic sci-Hi-C, sci-RNA-seq, and sci-ATAC-seq.

	XCI based on single cell interactions						
**dataset**	**total cells**	**XCI cells**	**B6/129 Xi cells**	**Spret/Cast Xi cells**	**XCI %**	**B6/129 Xi %**	**Spret/Cast Xi %**
**F121_d0**	986	116	59	57	12%	6%	6%
**F121_d3**	435	61	34	27	14%	8%	6%
**F121_d7**	243	107	68	39	44%	28%	16%
**F121_d11**	164	82	52	30	50%	32%	18%
**F121_NPC**	325	271	271	0	83%	83%	0%
**TsixStop_d0**	89	11	8	3	12%	9%	3%
**TsixStop_d3**	86	30	29	1	35%	34%	1%
**TsixStop_d7**	159	103	100	3	65%	63%	2%
**TsixStop_d11**	83	60	59	1	72%	71%	1%
**TsixStop_NPC**	975	806	806	0	83%	83%	0%
**replicate F121_d0**	393	49	27	22	12%	7%	6%
**replicate F121_d7**	47	17	17	0	36%	36%	0%
**replicate F121_d20**	89	66	44	22	74%	49%	25%
**replicate TsixStop_d0**	291	33	11	22	11%	4%	8%
**replicate TsixStop_d7**	51	16	11	5	31%	22%	10%
**replicate TsixStop_d20**	80	47	45	2	59%	56%	3%

	XCI based on single cell expression						
**dataset**	**total cells**	**XCI cells**	**129 Xi cells**	**Cast Xi cells**	**XCI %**	**129 Xi %**	**Cast Xi %**
**F121_d0**	486	111	68	43	23%	14%	9%
**F121_d3**	127	57	31	26	45%	24%	20%
**F121_d7**	76	65	51	14	86%	67%	18%
**F121_d11**	143	116	85	31	81%	59%	22%
**F121_NPC**	187	187	187	0	100%	100%	0%

	XCI based on single cell accessibiity						
**dataset**	**total cells**	**XCI cells**	**129 Xi cells**	**Cast Xi cells**	**XCI %**	**129 Xi %**	**Cast Xi %**
**F121_d0**	164	42	31	11	26%	19%	7%
**F121_d3**	702	239	159	80	34%	23%	11%
**F121_d7**	77	61	42	19	79%	55%	25%
**F121_d11**	174	153	100	53	88%	57%	30%
**F121_NPC**	178	170	169	1	96%	95%	1%

The determination of XCI status by each modality is described in the Methods section. For the sci-Hi-C data, the presence or absence of an Xi was determined by the long-range to mid-range difference (LMD) method on the allelic contact decay profiles (CDPs) of cells with at least 50 chr1 and chrX contacts. An LMD threshold value ($\square_{Xi}^{LMD}$) of 0.9, 0.9, 0.88, and 0.95 was used for F121, Tsix-stop, the F121 replicate experiment, and the Tsix-stop replicate experiment, respectively. For the sci-RNA-seq data, the total allelic expression difference threshold ($\square_{Xi}^{TAE}$) used was 1.21. For the sci-ATAC-seq data, the total allelic accessibility difference threshold ($\square_{Xi}^{TAA}$) used was 0.98.

Table S3. sci-RNA-seq cell counts and expression summary statistics.

sample	cells with >= 200 UMIs total expression			cells with >= 160 UMIs of expression per allele				
	cell count	mean UMIs	median UMIs	allelic cell counts	mean 129 UMIs	mean *cast* UMIs	median 129 UMIs	median *cast* UMIs
**F121_d0**	1,682	1,941	1,562	540	302	291	265	258
**F121_d3**	868	1,179	910	142	288	281	244	245
**F121_d7**	501	1,324	947	104	283	279	254	248
**F121_d11**	574	1,793	1,421	214	312	309	272	277
**F121_NPC**	497	2,509	2,158	344	344	342	302	318
**F123_d0**	1,654	1,923	1,538	503	295	275	263	240
**F123_d3**	646	902	626	73	295	265	273	247
**F123_d7**	1,102	1,210	915	181	276	253	250	221
**F123_d11**	925	1,415	1,028	206	302	279	264	242
**F123_NPC**	572	2,536	2,254	389	366	330	335	302

Counts of cells with at least 200 UMIs are given for each time point in the F121 sci-RNA-seq samples, along with mean and median UMIs per cell (see Fig. S6A–C). Also provided are the number of cells with at least 160 UMIs per allele for F121 sci-RNA-seq samples, along with mean and median UMIs per allele per cell (see Fig. S7A–D).

Table S4. sci-ATAC-seq cell counts and accessibility summary statistics.

sample	cells with >= 500 UMIs total accessibility			cells with >= 500 UMIs of accessibility per allele				
	cell count	mean UMIs	median UMIs	allelic cell counts	mean 129 UMIs	mean Cast UMIs	median 129 UMIs	median Cast UMIs
**F121_d0**	227	7,383	4,811	165	2,277	2,309	1,734	1,706
**F121_d3**	945	10,672	7,494	705	2,924	2,917	2,359	2,392
**F121_d7**	107	9,304	6,644	77	2,581	2,635	1,958	1,917
**F121_d11**	297	6,214	3,425	177	2,096	2,103	1,794	1,699
**F121_NPC**	234	8,327	6,143	180	2,186	2,190	1,779	1,758
**F123_d0**	140	9,383	7,091	114	2,579	2,492	2,287	2,213
**F123_d3**	153	12,123	9,756	119	3,415	3,353	2,913	2,891
**F123_d7**	457	6,320	3,947	293	2,006	1,960	1,485	1,488
**F123_d11**	167	4,054	1,777	70	1,874	1,776	1,179	1,183
**F123_NPC**	0	NA	NA	0	NA	NA	NA	NA

Counts of cells with at least 500 UMIs are given for each time point in the F121 sci-ATAC-seq samples, along with mean and median UMIs per cell (see Fig. S6D–F). Also provided are the number of cells with at least 500 UMIs per allele for F121 sci-ATAC-seq samples, along with mean and median UMIs per allele per cell (see Fig. S7E–H).

Table S5. MMD-MA parameters, cell downsampling and results.

		MMD-MA parameters			AUC scores					
		features	lambda 1	lambda 2	d0	d3	d7	d11	NPCs	mean
sci-RNA-seq vs sci-ATAC-seq	genome-wide	6	10^-3	10^-5	0.972	0.832	0.829	0.850	1.000	0.907
	autosomes	4	10^-3	10^-4	0.974	0.840	0.821	0.865	1.000	0.911
	chrX	3	10^-3	10^-5	0.677	0.547	0.516	0.499	0.905	0.650
	chr1	3	10^-7	10^-3	0.872	0.773	0.723	0.779	0.998	0.848
	chr1+chrX	3	10^-3	10^-5	0.904	0.750	0.732	0.821	0.999	0.863
sci-RNA-seq vs sci-Hi-C	genome-wide	5	10^-6	10^-7	0.779	0.694	0.717	0.727	0.788	0.759
	autosomes	3	10^-4	10^-7	0.687	0.727	0.738	0.673	0.616	0.684
	chrX	3	10^-4	10^-3	0.802	0.601	0.569	0.467	0.820	0.728
	chr1	3	10^-3	10^-3	0.761	0.526	0.441	0.314	0.699	0.648
	chr1+chrX	3	10^-7	10^-5	0.773	0.526	0.647	0.654	0.834	0.732
sci-ATAC-seq vs sci-Hi-C	genome-wide	3	10^-6	10^-6	0.773	0.699	0.621	0.617	0.639	0.683
	chrX	5	10^-4	10^-3	0.661	0.629	0.540	0.527	0.836	0.662

sci-RNA-seq vs sci-ATAC-seq	sci-RNA-seq						sci-ATAC-seq					
	d0	d3	d7	d11	NPC	total	d0	d3	d7	d11	NPC	total
original	404	98	73	164	285	1,024	165	705	77	177	180	1,304
subsampled	129	98	60	138	141	566	165	124	77	177	180	723
subsampled proportion	23%	17%	11%	24%	25%		23%	17%	11%	24%	25%	

sci-RNA-seq vs sci-Hi-C	sci-RNA-seq						sci-Hi-C					
	d0	d3	d7	d11	NPC	total	d0	d3	d7	d11	NPC	total
original	404	98	73	164	285	1,024	986	435	243	164	325	2,153
subsampled	404	98	73	78	154	807	849	206	153	164	325	1,697
subsampled proportion	50%	12%	9%	10%	19%		50%	12%	9%	10%	19%	

sci-ATAC-seq vs sci-Hi-C	sci-ATAC-seq						sci-Hi-C					
	d0	d3	d7	d11	NPC	total	d0	d3	d7	d11	NPC	total
original	165	705	77	177	180	1,304	986	435	243	164	325	2,153
subsampled	165	263	77	99	180	784	272	435	127	164	297	1,295
subsampled proportion	21%	34%	10%	13%	23%		21%	34%	10%	13%	23%	

MMD-MA parameters and area under the curve (AUC) scores for all the pairwise alignments reported in the study, along with the number of cells before and and after subsampling the cells from the five time-points (d0, d3, d7, d11, NPCs) to ensure that the fraction of cells in each time-point was consistent across the two datasets (see Methods).

Supplementary Video

Additional file 2. The Xi bipartite structure re-emerges after mitosis in Patski cells.
A movie compiled from aggregate allelic contact maps for the Xi and Xa (top row) and B6 and *spretus* chromosome 1 homologs (bottom row) using contact pairs from 64 mitotic, 512 early interphase, 1,032 mid interphase, and 2,615 late interphase cells, as grouped using the k-means clustering of the autosomal CDPs (Fig. S2D, E; see also Fig. 2K–N).
