# Supplementary material for: Single-cell landscape of nuclear configuration and gene expression during stem cell differentiation and X inactivation
Source: Genome Biol. 2021 Sep 27;22:279. doi: 10.1186/s13059-021-02432-w (PMC8474932; doi:10.1186/s13059-021-02432-w)
Supplement: Supplementary file 3 — Additional file 3: Review history. [file 13059_2021_2432_MOESM3_ESM.docx]

Review History

**First round of review**

**Reviewer 1**

**Are you able to assess all statistics in the manuscript, including the appropriateness of statistical tests used?**

Yes, and I have assessed the statistics in my report.

**Comments to author:**

Within the manuscript "Single-cell landscape of nuclear configuration and gene expression during stem cell differentiation and X inactivation", by Bonora et al, the authors applied a single-cell epigenetic profiling approach, by means of scRNA-seq, scHi-seq and scATAC-seq, to obtain insight into early differentiation and X inactivation (XCI) of mouse embryonic stem cells (mESCs). The authors make interesting observations, such as that the formation of the bipartite inactivated X (Xi) chromosomal topology is delayed as compared to silencing and inaccessibility of the Xi. This topological changes of the Xi towards the bipartite structure appears to be a binary switch, rather than a progressive change from an open towards the bipartite structure. The authors thoroughly put the events associated with XCI in perspective of general changes that occur during differentiation of mESCs. This enables the authors to observe that next to the Xi, also the autosomes and the active X chromosome (Xa) show an increase in large-range interactions during differentiation, an exciting finding.

Although the manuscript is highly descriptive, the authors managed to generate an interesting and diverse dataset which is well analyzed. Also, the manuscript is very well written and nicely illustrated with informative figures. Having said that, the figures represent a high level of analysis and therefore remain somewhat abstract, which might make the paper challenging for non-experts. I would have appreciated somewhat more "raw" data, like the contact maps of figures 2A, E, K, and M, or other plotted structures of the autosomes and Xi or Xa; Similar: figure 3 fully consists of a large range of similar-looking, somewhat abstract graphs; any efforts to make these more attractive, accessible and comprehensive is beneficial for readers.

With respect to content, there is a range of choices and conclusion that might need additional explanation or reconsideration:

Major concerns:

1. A large part of the manuscript is dedicated to revealing how the bipartite Xi is being formed from an Xa. The setup of this study is well designed to address this issue, as heterogeneity between cells is captured in the single cell analysis. However, I do miss an analysis where the authors include all cells of the differentiation time course to make an unbiased pseudotime analysis of the topological changes that occur on the Xi specifically. Such analysis should have the potential to capture the dynamics going from an Xa to the bipartite Xi, and might be able to identify "hotspots" on where folding towards the Xi initiate. Indeed, the authors conclude "While we cannot exclude the existence of intermediate structures undetectable by our approach our single cell", but I feel such alternative approaches might provide additional insight on important X-linked elements that drive the formation of the bipartite Xi. Indeed, the Xi (red line) in Fig 3a bottom panel (day 0 to 11 to MPCs) seem to show a gradual increase of long-range interactions over time, supportive of a more gradual model of Xi bipartite formation.

2. Also for the scRNA-seq and scATAC-seq, it would be highly informative to do an (integrated) pseudotime analysis specifically on the Xi, and subsequently investigate the dynamics of gene silencing /accessibility in relation to features of the X chromosome. This could also shed light on the relationship between accessibility and gene silencing for individual genes on the Xi.

3. I am not convinced by the data that lead the authors to conclude "A rapid structural change takes place at mitosis" (line 168). This conclusion is based on as few as 21 cells (Fig 2K, L) or 64 cells (Additional file 2). Fig S1a shows that any topological structures are hardly visible if using low cell numbers. Also, the way mitotic cells are identified is indirect. I feel that if the authors would like to draw solid conclusions on this topic, they should include independent evidence. For example, the use of a FUCCI cell cycle reporter which is available in mESCs would allow to sort cells based on cell cycle, after which more bulk type assays could be performed.

4. The authors conclude that they observe three different topologies, an early, an intermediate and a differentiated topology (Figure 4A). However, could it be that they observe a branch that is more associated with endo- and mesoderm ("the intermediate branch"), while the other branch ("the differentiated branch") more consists of ectoderm (/neural)-like cells? Such ectoderm (/neural)-like cells could be over-represented in the D11 EBs. Could this be an alternative explanation? Also, it would be informative to get more insight in the "intermediate" and "differentiated" branch, besides that the number of short- and long-range interactions change. Where are there interactions located? Are these associated with specific genomic elements? The authors have all data in hand to provide comprehensive insight into such questions.

5. Figure 4C shows a separate branch of cells in between the early and differentiated branch, which is absent in Figure 4A or 4B. What does this branch represent?

**Reviewer 2**

**Are you able to assess all statistics in the manuscript, including the appropriateness of statistical tests used?**

Yes, and I have assessed the statistics in my report.

**Comments to author:**

In this manuscript Bonora et al. present a systematic study of differentiating mouse ESC and X inactivation based on scRNA-seq, scATAC-seq and scHI-C measurement across times.

The manuscript is overall well written and there are several novel potential findings. New computational methodologies (some not yet published but with available preprints) are utilized to integrate the three modalities.

I praise the authors for sharing pre-publication all the datasets used in this manuscript and the computational procedures.

Major points

1) I have some concerns about the interpretation of the results. First some of the computational procedures have not been independently validated and/or extensively tested on novel inputs (for example the CDP) and compared with other procedures already published.

How different would be the conclusions by using for example the integration method proposed in Seurat 4 instead of MMD-MA or a different trajectory analysis instead of Monocle2 (for example Slingshot?). I want to stress that I am not asking for an extensive comparison with other available methods rather a parallel confirmation by other well-established procedures.

2) Please discuss how batch effects may influence your results especially for the integration procedure or for the observed delay experienced by female cells compared to male cells (you could consider to use Harmony).

3) "even though some cells from different time points were dispersed across all three branches, indicative of variability in nuclear structure" How can we exclude this is just not an artifact of the normalization used or batch effects? Similarly, given the sparsity of these assays, how can we exclude that cells without the ultra-long range interactions have poor coverage? Can you color the cells in this plot by their "contact score"?

4) "A majority of cells (56 - 65%) in the trajectories cluster at the terminal ends of each branch, which suggests the existence of three distinct types of nuclear structure as defined by CDPs" Based on this trajectory analysis cells seem to have a continuous pseudotime and not forming disconnected components. Is this an artifact of the trajectory analysis ? How stable is this analysis to the number of CDP selected ? How the same features will look like in a UMAP plot similar to the one presented in Fig 1D?

5) "To our knowledge this is the first time an in silico co-assay has been used to align three disjoint single cell datasets" The proposed integration method seems powerful, however the current visualization allows to consider only two assays at the same time. Would it be possible with this method to have a visualization based on a shared space for all the three datasets?

6) "This multimodal alignment greatly improved clustering of cells to identify single-cell relationships among gene expression, chromatin accessibility, and nuclear structure during differentiation." I am not sure about the meaning of clustering in this sentence since no clustering was performed.

Minor Points

1) Some figures are really dense and could be reorganized to simplify the presentation. For example, I suggest trying to use heatmaps to aid the visual comparison across of the panels in Fig 3A,B,C.

2) Line 245, there is an extra "."

**Authors Response**

**Point-by-point responses to the reviewers’ comments:**

Reviewer #1

*We thank the reviewer for their kind comments and careful consideration of our manuscript.*

General concern:

I would have appreciated somewhat more "raw" data, like the contact maps of figures 2A, E, K, and M, or other plotted structures of the autosomes and Xi or Xa; Similar: figure 3 fully consists of a large range of similar-looking, somewhat abstract graphs; any efforts to make these more attractive, accessible and comprehensive is beneficial for readers.

*We agree that alternative representations of the data in Fig. 3 will aid the readers and provide some variety. We have taken the following steps to address this concern:*

*1.	We broke up the original Fig. 3 into two separate figures in order to include additional panels in both. The new Fig. 3 now focuses on the Tsix-stop time course experiment, and the new Fig. 4 shows the results of the F121 experiment, as well as a comparison of the two time course experiments and additional raw data in the form of contact maps.*

*2.	We added heatmaps of Tsix-stop allelic contact decay profiles (CDPs) like those in Fig. 2B, but for the time course, as new Fig. 3A, and allelic CDP heatmaps for F121 in new Fig. S3C. These plots emphasize the presence of a mixture of cells with and without an Xi bipartite structure during differentiation.*

*3.	We moved existing F121 and Tsix-stop pseudobulk contact maps from the supplementary Fig. S4A, B to new main Fig. 4F, G, and changed their color scheme in order to contrast this time course data and the Patski data. F121 and Tsix-stop pseudobulk contact maps were also added to Fig. S2F, H in order to highlight changes to chromosome structure during the cell cycle. The actual “raw” sci-Hi-C contact matrices are sparse, as indicated by the contact map in Fig. S2A, and are not very informative on their own. But pseudobulk contact maps reveal 3D conformation structure, as shown in Fig. S1, using an increasing proportion of Patski cells. See also Fig. 2A, E, K and N. In order to obtain sufficient numbers of cells for visually informative contact maps, we combined data from both F121 and Tsix-stop cells for the new Fig. 4F, G.*

Major concerns:

1.	A large part of the manuscript is dedicated to revealing how the bipartite Xi is being formed from an Xa. The setup of this study is well designed to address this issue, as heterogeneity between cells is captured in the single cell analysis. However, I do miss an analysis where the authors include all cells of the differentiation time course to make an unbiased pseudotime analysis of the topological changes that occur on the Xi specifically. Such analysis should have the potential to capture the dynamics going from an Xa to the bipartite Xi, and might be able to identify "hotspots" on where folding towards the Xi initiate.

*We agree that this type of analysis would be informative. However, X-chromosome inactivation (XCI) is random during the differentiation of F121 cells, so we do not know which allele is destined to be the future Xi in the earlier time points. We can only assess the XCI status of cells after the onset of XCI and only for cells with sufficient allelic coverage. Unfortunately, for the Tsix-stop line – where we do know which allele will be subject to XCI because cells have been engineered to have XCI skewed towards their 129 allele – we did not have sufficient early time points cells to construct an informative trajectory and were unable to generate additional Tsix-stop cells in time for resubmission. This is an avenue we intend to follow-up on.*

Indeed, the authors conclude "While we cannot exclude the existence of intermediate structures undetectable by our approach", but I feel such alternative approaches might provide additional insight on important X-linked elements that drive the formation of the bipartite Xi. Indeed, the Xi (red line) in Fig 3a bottom panel (day 0 to 11 to MPCs) seem to show a gradual increase of long-range interactions over time, supportive of a more gradual model of Xi bipartite formation.

*Although the plots in the original Fig. 3 (now new Fig. 3 and 4) can be interpreted to indicate a gradual model for Xi formation, this could be due to a gradual change in the proportion of cells having acquired the Xi 3D structure, while the actual switch in conformation might well be more abrupt, as indicated by the pseudotime-related analysis in original Fig. 4 (now new Fig. 5). In particular, new Fig. 5E, F show that cells tend to cluster towards the ends of each branch, suggesting three distinct states rather than a continuous change in 3D genomic structure during development (see manuscript Results section 3, lines 283 – 291). The allelic CDP heatmaps in the new Fig. 3A and S3C – where the CDPs of individual cells are more clearly visible that in the CDP plots – support the notion that the conformation change is likely quite abrupt. Rather, it is the proportion of cells that show the conformational change that is gradual, as does the comparison of CDPs for cells at the distal ends of each of the three branches (Fig. 5G, H; Fig. S5F).*

2.	Also for the scRNA-seq and scATAC-seq, it would be highly informative to do an (integrated) pseudotime analysis specifically on the Xi, and subsequently investigate the dynamics of gene silencing /accessibility in relation to features of the X chromosome. This could also shed light on the relationship between accessibility and gene silencing for individual genes on the Xi.

*As explained above, since F121 is not a skewed system, we do not know which allele is the future Xi and cannot track Xi-specific cell expression and accessibility in the F121 cells. We therefore cannot build Xi-specific trajectories based on these F121 data.*

*We did attempt to compare accessibility and expression trends (e.g. % and location of Xi genes that have lost accessibility or expression), but our data was too sparse to confidently observe such trends for individual genes at the single cell level.*

3.	I am not convinced by the data that lead the authors to conclude "A rapid structural change takes place at mitosis" (line 168). This conclusion is based on as few as 21 cells (Fig 2K, L) or 64 cell (Additional file 2). Fig S1a shows that any topological structures are hardly visible if using low cell numbers. Also, the way mitotic cells are identified is indirect. I feel that if the authors would like to draw solid conclusions on this topic, they should include independent evidence. For example, the use of a FUCCI cell cycle reporter which is available in mESCs would allow to sort cells based on cell cycle, after which more bulk type assays could be performed.

*Our strategy of using non-allelic CDPs to determine cell cycle is borrowed from Nagano et al. [1], who generated single cell Hi-C data on cells at different stages of the cell cycle and showed that one can infer cell cycle state from their genome-wide CDPs. However, we are in agreement with the reviewer that one cannot attribute rates of changes to cell cycle states without using a cell cycle reporter. We therefore no longer make any claim about the speed of the structural changes in the manuscript.*

*We also agree that the findings we presented using Patski cell data would benefit from additional support in the form of data from other cell lines. We have therefore added equivalent analysis for an additional differentiated cell type where XCI is skewed towards one allele, namely Tsix-stop and F121 neural precursor cells (NPCs), where the 129 allele is the inactive X chromosome (Xi) (see manuscript Results section 2, lines 182 – 192; updated Fig. S2F, G). As we saw for Patski cells, these plots show that the Xi-specific bipartite structure and distinctive CDP is lost in mitotic cells and re-emerges during interphase. The same analysis was also conducted on Tsix-stop and F121 embryonic stem cells (ESCs) to show that the typical compartmental structure is also lost in this cell type during mitosis only to re-emerge during interphase, but neither allele takes on the Xi bipartite structure since XCI has yet to occur in ESCs (see manuscript Results section 2, lines 182 – 192; updated Fig. S2H, I).*

*We have also added the observation that, for a small proportion of differentiated cells, the inability to detect a bipartite X-chromosome is due to the fact that those cells are undergoing mitosis (see manuscript Results section 1, lines 152 – 158; section 2, lines 210 – 214; section 2, lines 225 – 229). In most cases, however, the apparent lack of an Xi 3D conformation is likely due to a combination of variance in the inherent nature of the structure between different single cells and variance in the ability of the Hi-C assay used to assess it.*

4. The authors conclude that they observe three different topologies, an early, an intermediate and a differentiated topology (Figure 4A). However, could it be that they observe a branch that is more associated with endo- and mesoderm ("the intermediate branch"), while the other branch ("the differentiated branch") more consists of ectoderm (/neural)-like cells? Such ectoderm (/neural)-like cells could be over-represented in the D11 EBs. Could this be an alternative explanation?

*Based on the literature, EBs derived from fetal bovine serum (FBS) are mesodermal in nature [2,3], whereas NPCs are ectodermal. And yet a majority of d11 EBs and NPCs are found together on the late branch of the sci-Hi-C CDP trajectory (updated Fig. 5A – C). This observation argues against the branches being driven by the formation of different germ layers. In order to confirm the mesodermal character of the F121 EB cells, we performed topic modeling and gene ontology (GO) analysis on non-allelic the F121 sci-RNA-seq and sci-ATAC-seq count matrices. We have included the results of this analysis as a main figure (new Fig. 7), which includes heatmaps (new Fig. 7A, E), PCA projections, and UMAPs (new Fig. 7B, F). These results clearly show the d7/d11 F121 cells and NPCs to be well separated, implying that they have very distinct natures. This is largely confirmed by GO analysis conducted on topics associated with d7/d11 cells and NPCs, albeit a small subset of d11 cells show some neuronal characteristics (see manuscript Results section 3, lines 278 – 289 and section 4, lines 379 – 397; 438 – 441). Further evidence against mesoderm cells being exclusively associated with the intermediate branch and the ectodermal cells with the differentiated branch is that the same three-branch sci-Hi-C CDP trajectory is obtained when NPCs are omitted from the analysis (updated Fig. S5A; see manuscript Results section 3, lines 272 – 277). In contrast, when NPCs are omitted from the sci-RNA-seq and sci-ATAC-seq trajectory analysis, a single developmental arc is obtained (updated Fig. 6H; I; see manuscript Results section 4, lines 363 – 367). We therefore conclude that that the CDP trajectories do represent nuclear configuration changes that are associated with differentiation rather than cell types (see manuscript Discussion, lines 568 – 573).*

Also, it would be informative to get more insight in the "intermediate" and "differentiated" branch, besides that the number of short- and long-range interactions change. Where are there interactions located? Are these associated with specific genomic elements? The authors have all data in hand to provide comprehensive insight into such questions.

*This is a great question and one that we also have. We would have to look for enriched contacts at each interaction length scale and then to look for associations with linear genomic features in those regions. We would like to pursue this question in a follow up study.*

5. Figure 4C shows a separate branch of cells in between the early and differentiated branch, which is absent in Figure 4A or 4B. What does this branch represent?

*That branch is likely due to the lower signal-to-noise ratio when running the trajectory analysis on just the X chromosome. Three connected sub-branches can be considered to be part of the early branch since most cells were d0 cells. This has been more clearly stated in the legend to updated Fig. 5C, and we have added a table showing the counts per time point along each of the branches before and after collapsing three early sub-branches into a single branch (new Fig. S5C). Small sub-branches were also obtained for the genome-wide CDP trajectory where NPCs were omitted (updated Fig. S5A). In this case, we collapsed two early and two differentiated sub-branches into single early and differentiated branches, respectively, something that we have now described more clearly in a new table (Fig. S5B).*

Reviewer #2

*We thank the reviewer for their constructive comments and careful reading of our manuscript.*

Major points

1) I have some concerns about the interpretation of the results. First some of the computational procedures have not been independently validated and/or extensively tested on novel inputs (for example the CDP) and compared with other procedures already published.

*Yes, the use of trajectory analysis on CDP data is novel, and we have now emphasized this in the discussion (lines 553 – 554). However, CDP analysis is commonly used in the field [1] and is particularly useful in sc-Hi-C analysis because it helps mitigate the effects of sparsity in the data.*

How different would be the conclusions by using for example the integration method proposed in Seurat 4 instead of MMD-MA or a different trajectory analysis instead of Monocle2 (for example Slingshot?). I want to stress that I am not asking for an extensive comparison with other available methods rather a parallel confirmation by other well-established procedures.

*We did provide corroborating trajectories based on an unsupervised alternative approach that does not rely on pre-defining differential elements, namely topic modeling (Figs. S6G – I and S8A – F; see manuscript Results section 3, lines 272 – 274; section 5, lines 479 – 484).*

*As suggested, we also tried running Slingshot [4] trajectory analysis on our data (Fig. R1). The trajectories obtained when using non-allelic F121 and F123 sci-RNA-seq data look very similar to those obtained by Monocle2’s DDRTree algorithm, although the cells were not iteratively moved closer to the derived tree structure so look more dispersed (Fig. R1A; updated Fig. 6C). However, a similar tree structure is obtained, with NPCs forming their own branch away from the default EB developmental pathway. Furthermore, female cells also appeared to be delayed in their development relative to male cells in the Slingshot trajectory. Indeed, when one omits NPCs from the trajectory analysis based on F121 single cell expression, one obtains an unbranched developmental arc from d0 to d11 cells similar to that obtained using DDRTree, and male cells precede female cells along that arc (Fig. R1B; updated Fig. 6F). In addition, the Slingshot trajectory based on the d0 to d11 F121 single cell accessibility data produces a similar unbranched trajectory to that seen using DDRTree, with male cells preceding female along the developmental arc (Fig. R1C; updated Fig. 6G). We also applied Slingshot to our allelic sci-Hi-C CDP data but with limited success.*

*With regards to using Seurat 4 instead of MMD-MA, in the supplementary methods of the paper [5] it states that the weighted nearest neighbor procedure works on datasets where measurements are taken from the same cell (i.e., co-assay data). "The weighted nearest neighbor (WNN) procedure implemented in Seurat v4 is designed to integrate multiple types of data that are collected in the same cells to define a single unified representation of single-cell multimodal data." For instance, for the CITE-Seq data they use in the paper, they use two modalities – RNA and protein measured in each single cell. The "unsupervised" term appears to refer to the fact that these "data points are unlabeled" and so one does not have information about the state of the cells. The paper states that "Cell annotations are derived from WNN analysis, and reveals heterogeneity within T cells and progenitors that cannot be discovered by either modality independently." For other cases where measurements were not from the same cell, they use the anchor-based integration of Seurat v3: "To facilitate the identification of shared cell types across datasets, we applied our previously developed ‘anchor’ workflow to integrate the datasets.” This rules out aligning sci-Hi-C data with either of the other two data types because with Seurat one can only align the sci-RNA-seq and the sci-ATAC-seq since the method requires datasets with a shared feature space or the ability to learn such a shared feature space. This would be hard to define for sci-Hi-C data with respect to sci-RNA-seq and sci-ATAC-seq. Because Seurat V4 is not a fully unsupervised method in the same way that MMD-MA is, Seurat V4 was not compared to MMD-MA, but MMD-MA has been compared to other unsupervised approaches, namely JLMA, UnionCom, and PCA [6].*

2) Please discuss how batch effects may influence your results especially for the integration procedure or for the observed delay experienced by female cells compared to male cells (you could consider to use Harmony).

*F121 and F123 were processed together (cell culture, harvest, and sci-omics experiments) to minimize potential batch effects between them so batch normalization would not be needed. To confirm this, we applied Monocle3’s [7] implementation of Batchelor [8] to our sci-RNA-seq data, treating the sexes as two separate batches. UMAPs of the data before (new Fig. 6F) and after (Fig. R2A) the application of the algorithm shows that cells cluster in a similar manner in both cases and that a distinct separation between the female and male cells is not lost after treating the sexes as two separate batches (see manuscript Results section 4, lines 356 – 361). Furthermore, the elevated X:A ratio seen for ESCs (d0) and d3 female persists (new Fig. 6G; Fig. R2B; see manuscript Results section 4, lines 361 – 363). Trajectory analysis performed on the batch corrected data produces essentially the same tree structure as that obtained on the uncorrected data, with female cells also delayed in their development relative to male cells (Fig. 6C; R2C). And when NPCs are omitted, one obtains the same unbranched developmental arc from d0 to d11 cells similar to that obtained using uncorrected data, with male cells preceding female cells along that arc (updated Fig. 6H; Fig. R2D). Taken together, these results support our contention that batch correction between F121 and F123 cells is not necessary and does not change the results when applied, and that the sex differences we observe are real and lend support to previous observations of this phenomenon (see manuscript Results section 4, lines 354 – 377; Discussion, lines 608 – 610).*

3)	"even though some cells from different time points were dispersed across all three branches, indicative of variability in nuclear structure" How can we exclude this is just not an artifact of the normalization used or batch effects? Similarly, given the sparsity of these assays, how can we exclude that cells without the ultra-long range interactions have poor coverage? Can you color the cells in this plot by their "contact score"?

*For the allelic CDP analysis, we only considered Patski cells with at least 100 intrachromosomal contacts from each allele (chromosome) and cells with at least 50 intrachromosomal contacts from each allele for all other cell types. CDP counts were then normalized (z-scaled) to account for coverage differences between cells. This was described in the legends of related figures, and we have now described this in the Methods section as well (see lines 696 – 697). To verify that these steps did mitigate the influence of coverage in the CDP trajectory analysis, we have now included a plot of the genome-wide trajectory colored by the total contact count for each cell, which shows that the branch structure is not driven by coverage differences (new Fig. S5D; see manuscript Results section 3, lines 289 – 291). To show this in a more quantitative manner, we now also include cumulative distribution functions of the total contact counts of cells along each branch, which are relatively similar to one another (new Fig. S5E).*

4)	"A majority of cells (56 - 65%) in the trajectories cluster at the terminal ends of each branch, which suggests the existence of three distinct types of nuclear structure as defined by CDPs" Based on this trajectory analysis cells seem to have a continuous pseudotime and not forming disconnected components. Is this an artifact of the trajectory analysis? How stable is this analysis to the number of CDP selected? How the same features will look like in a UMAP plot similar to the one presented in Fig 1D?

*Although some cells are distributed along the branches of the trajectory, a high proportion of cells are found at the distal ends of each branch, a pattern that we show in Fig. S5B. To better highlight this pattern, we have now moved this panel to a main figure (updated Fig. 5E). In addition, we have added plots of the cumulative distributions of the proportion of cells per branch with pseudotime normalized between 0 and 1 along each of them (new Fig. 5F). The plots are annotated with vertical lines that cross inflection points in the cumulative distribution beyond which over 56% of the cells are seen to accumulate. This data highlights that the majority of the cells are disconnected in that space. The inflection points serve as the thresholds beyond which cells are considered to be distal cells along each branch. The finding that the cells at the distal ends of each of the three branches have very distinctive CDPs (originally Fig 4D, E; updated Fig 5G, H) provides further support for the claim that the trajectory analysis separates the cells into disjoint components based on real differences in their 3D conformation (see manuscript Results section 3, lines 283 – 288). This observation suggests the existence of three distinct types of nuclear structure as defined by allelic CDPs. In addition, in the heatmaps presented in updated Fig. S3C, differences between the allelic CDPs of individual cells are clearly visible, which provides additional support for the existence of cells with distinct nuclear conformation.*

*Similar trajectories are obtained when one uses CDPs from all chromosomes (genome-wide trajectories), only autosomes, only the X-chromosome, and when NPCs are not included, which speaks to the consistency of the trajectory analysis based on CDPs (updated Fig. 5A – C; updated Fig. S5A – C; see manuscript Results section 3, lines 270 – 277), and which we believe is indicative of the overarching trends in the trajectories that we obtain. Although trajectories with more than three branches can arise, depending on the number of differential CDP bins used, the additional branches tend to be superficial sub-branches. These sub-branches can be considered to be part of a main branch, such as the case with the trajectories based on X-chromosomal CDPs only or when NPCs are omitted (updated Fig 5C; updated Fig. S5A). We have now added tables with explanations in the legend to clearly show how sub-branches collapse (new Fig. S5B, C). The sub-branches are likely due to the lower signal-to-noise ratio when running the trajectory analysis using less data.*

*PCA projections and UMAP of the allelic CDPs were provided in Fig. S8C and F, respectively, albeit based on topic matrices generated from the allelic CDP count matrices (see manuscript Results section 3, lines 272 – 274; section 5, lines 479 – 484). And we have now added text in section 3 to emphasize this within the context of the description of the allele CDP trajectory results. Although these projections lack a tree structure, they do show that the cells co-localize by time point and are somewhat disjoint. The utility of using the DDRTree trajectory approach for this analysis is that it facilitated their separation into branches, with the majority of the cells at the terminal ends of each branch. We acknowledge that the DDRTree algorithm is a) a semi-supervised method based on differential features between time points and b) that it is somewhat aggressive in the way that it aligns cells to the inferred tree structure, but this serves the purpose of highlighting what we believe to be real changes in the 3D conformations of the cells during development. The differences between the CDPs of cells at the distal ends of each branch are highlighted in updated Fig. 5E – H and updated Fig. S5F.*

5)	"To our knowledge this is the first time an in silico co-assay has been used to align three disjoint single cell datasets" The proposed integration method seems powerful, however the current visualization allows to consider only two assays at the same time. Would it be possible with this method to have a visualization based on a shared space for all the three datasets?

*This is something we have considered and started looking into, but it will take much more work and time and thus is not included in this study.*

6)	"This multimodal alignment greatly improved clustering of cells to identify single-cell relationships among gene expression, chromatin accessibility, and nuclear structure during differentiation." I am not sure about the meaning of clustering in this sentence since no clustering was performed.

*We meant “clustering” in a more visual and less technical sense in that they co-localized or group together. We have now reworded this more carefully (see manuscript Results section 5, line 504).*

Minor Points

1)	Some figures are really dense and could be reorganized to simplify the presentation. For example, I suggest trying to use heatmaps to aid the visual comparison across of the panels in Fig 3A,B,C.

*We thank the reviewer for their suggestion and agree that the use of heatmaps is beneficial and will provide some necessary variety. Please see our opening response to Reviewer 1 for a description of the changes we made to Fig 3.*

2)	Line 245, there is an extra "."

*Corrected.*

*Figure R1. Trajectory analysis of F121 and F123 non-allelic sci-RNA-seq and sci-ATAC-seq data using Slingshot.*

*A)	As in Fig. 6C, but using slingshot.*

*B)	As in FIg. 6H, but using slingshot.*

*C)	As in FIg. 6I, but using slingshot.*

*Figure R2. Delay in the onset of differentiation in female cells relative to male cells is still clearly visible even after batch correction for sex.*

*A)	As in Fig. 6F UMAP projection of count matrix generated for non-allelic F121 and F123 sci-RNA-seq data, but after batch correction for sex using the Monocle3 implementation of Batchelor. In the left plot each data point represents a cell colored by time point and on the right cells are color by sex.*

*B)	As in Fig. 6G, but after batch correction using the Monocle3 implementation of Batchelor. Cells are colored by the ratio of X-chromosomal to autosomal gene expression (X:A ratio) based on genes that were expressed in at least 10% of cells.*

*C)	As in Fig. 6A, but after applying batch correction for sex and faceted by cell sex (F121 on the left, F123 on the right).*

*D)	As in Fig. 6H, but after applying batch correction for sex and with trajectory faceted by cell sex (F121 on the left, F123 on the right). Below are plots of cumulative distributions of cells’ pseudotimes (rescaled between 0 and 1) along the trajectory arc for female F121 and male F123 cells.*

*References*

*1.	Nagano T, Lubling Y, Várnai C, Dudley C, Leung W, Baran Y, et al. Cell-cycle dynamics of chromosomal organization at single-cell resolution. Nature. 2017;547:61–7.*

*2.	Keller GM. In vitro differentiation of embryonic stem cells. Curr Opin Cell Biol. 1995;7:862–9.*

*3.	Argelaguet R, Clark SJ, Mohammed H, Stapel LC, Krueger C, Kapourani C-A, et al. Multi-omics profiling of mouse gastrulation at single-cell resolution. Nature. 2019;576:487–91.*

*4.	Street K, Risso D, Fletcher RB, Das D, Ngai J, Yosef N, et al. Slingshot: cell lineage and pseudotime inference for single-cell transcriptomics. BMC Genomics. 2018;19:477.*

*5.	Hao Y, Hao S, Andersen-Nissen E, Mauck WM, Zheng S, Butler A, et al. Integrated analysis of multimodal single-cell data [Internet]. bioRxiv. 2020 [cited 2021 Apr 30]. p. 2020.10.12.335331. Available from: https://www.biorxiv.org/content/10.1101/2020.10.12.335331v1.abstract*

*6.	Singh R, Demetci P, Bonora G, Ramani V, Lee C, Fang H, et al. Unsupervised manifold alignment for single-cell multi-omics data. Proceedings of the 11th ACM International Conference on Bioinformatics, Computational Biology and Health Informatics. New York, NY, USA: Association for Computing Machinery; 2020. p. 1–10.*

*7.	Cao J, Spielmann M, Qiu X, Huang X, Ibrahim DM, Hill AJ, et al. The single-cell transcriptional landscape of mammalian organogenesis. Nature. 2019;566:496–502.*

*8.	Haghverdi L, Lun ATL, Morgan MD, Marioni JC. Batch effects in single-cell RNA-sequencing data are corrected by matching mutual nearest neighbors. Nat Biotechnol. 2018;36:421–7.*

**Second round of review**

**Reviewer 1**

I feel the authors addressed the comments from the reviewers well. Of note, the paper is very technical and thereby somewhat difficult to read. It is also lengthy (for example, 9 main figures), probably partly due to the additional analysis requested by the reviewers. I feel that any attempts to shorten the manuscript and make it more accessible, as well as to reduce the number of figures, would benefit the readers. Otherwise, the current manuscript represent very solid and interesting work, and I fully support acceptance.

**Reviewer 2**

The authors have thoroughly and satisfactorily addressed my comments.
